# Using normalisation process theory to identify factors facilitating the scaling up of parenting programs for immigrant parents

**DOI:** 10.3389/fpsyg.2024.1456586

**Published:** 2024-12-04

**Authors:** Fatumo Osman, Ulla-Karin Schön, Raziye Salari

**Affiliations:** ^1^School of Health and Welfare, Dalarna University, Falun, Sweden; ^2^Department of Social Work, Stockholm University, Stockholm, Sweden; ^3^Department of Psychology, Friedrich-Alexander-Universität Erlangen-Nürnberg, Erlangen, Germany; ^4^Child Health and Parenting (CHAP), Department of Public Health and Caring Sciences, Uppsala University, Uppsala, Sweden

**Keywords:** culturally tailored, immigrants, normalization process theory, parents, parenting programs, sustainable implementation, scaling up

## Abstract

**Objective:**

As most studies focus on the initial adaptation of culturally tailored parenting programs in real-world settings, scalability and sustainability remain understudied. In this study, we used normalization process theory (NPT) as a conceptual framework to identify and understand the contextual factors impacting the scalability and sustainability of a culturally tailored parenting program, Ladnaan, in three Swedish municipalities.

**Method:**

Nineteen managers, coordinators, and group leaders completed a survey and participated in individual or focus group interviews.

**Results:**

Our analyses showed that participants agreed on the need for the Ladnaan intervention among immigrant families. However, they were concerned that insufficient resources may hinder the recruitment and retention of parents, particularly as certain resources were allocated to the delivery of other, non-evidence-based interventions. Both managers and coordinators emphasized that embedding Ladnaan into everyday practice is conditioned on the collective work and engagement of all stakeholders, which will occur only if local authorities feel they own the program. The recruitment and retention of group leaders was mentioned as a key challenge in sustaining Ladnaan as the need for the program fluctuated over time.

**Conclusion:**

These findings highlight the importance of promoting evidence-based programs within the services available for forcibly displaced parents, and of coordinating efforts to scale up and sustain these programs.

## Introduction

Given the sharp increase in immigration, supporting forcibly displaced families has become a public health issue in European countries such as Sweden (Eurenius, 2019). Parents who have been forcibly displaced bring a sets of skills and resources to their parenting in their new home country. However, they are also vulnerable to encountering various challenges regarding their parenting and adjustment to the new country (Baghdasaryan et al., 2021; Bergnehr, 2018; Osman et al., 2016). They have reported that adjusting to the new country causes acculturation stress, which affects their confidence in parenting (Bergnehr, 2018; Mangrio et al., 2020; Osman et al., 2016; Västhagen et al., 2022). Studies have also shown that problems in the parent–child relationship occur due to acculturation gaps between parents and children (Bergnehr, 2018; Osman et al., 2021a). Offering targeted and culturally tailored parenting support programs can help eradicate health and social inequalities among forcibly displaced parents families (Gillespie et al., 2022; Sanders et al., 2022). It is well documented that evidence-based parenting programs strengthen parent–child relations, particularly programs offered to forcibly displaced families (Hamari et al., 2022). Parenting programs tailored to forcibly displaced parents have been shown to have a positive impact on both children’s and parents’ outcomes (Bjorknes and Manger, 2013; Lee and Kim, 2022; Osman et al., 2019; Osman et al., 2017a,b; Renzaho and Vignjevic, 2011). However, such programs are not widely available or scalable (Gillespie et al., 2022; Sanders et al., 2022).

Most studies on the implementation of culturally tailored parenting programs have focused on the *adoption* and *implementation* of these programs in real-world settings, but the *scalability* and *sustainability* of evidence-based programs in health care and social services remain understudied (Christensen and Prout, 2002; Parra-Cardona et al., 2023; Sanders et al., 2022). This is particularly the case regarding the scalability of parenting programs for forcibly displaced populations (Hodge and Turner, 2016). In this study, we want to identify and understand the factors impacting the scalability and sustainability of the Ladnaan intervention in Swedish municipalities using NPT. Existing studies on parenting programs have explored the factors contributing to their scalability and sustainability in general and across various settings. However, only a few have examined the potential for scaling up and sustaining programs tailored to migrant and ethnic populations (Apers et al., 2023; Brotman et al., 2021; Hodge and Turner, 2016; Hodge et al., 2017). Integrating and sustaining parenting programs for disadvantaged groups including immigrant parents into regular services requires the coexistence of three main factors (Hodge and Turner, 2016). First, it is crucial that the planned program is known to be effective and easy to implement for staff in their everyday practice (Hodge and Turner, 2016; Hodge et al., 2017). A recent study suggests that nonspecialist facilitators and peer-led supervision can enhance long-term commitment to a program and reduce the loss of expertise due to staff shifting tasks or changing jobs (Sanders et al., 2022). The second main factor is that the organization in which the program is implemented must support the implementation and the process to scale it and, most importantly, have a coordinator in place to manage the implementation (Hodge and Turner, 2016; Hodge et al., 2017). A systematic literature review conducted in Sweden demonstrated that a coordinator to facilitate the implementation work is crucial for the sustainability and scalability of parenting support programs (Ericsson and Sarkadi, 2021). The third main factor that facilitates the scalability and sustainability of parenting programs is collaboration between various stakeholders and the community in general (Ericsson and Sarkadi, 2021; Hodge and Turner, 2016; Hodge et al., 2017). Moreover, the implementing organization needs to possess the ambition to scale up, make the program sustainable, and not rely on project-based implementation, which means securing the economic resources necessary for scalability and sustainability (Ericsson and Sarkadi, 2021; Sanders et al., 2022).

### Framework for the implementation of interventions

Normalization process theory (NPT) explains how complex interventions are embedded and incorporated into routine practices (May and Finch, 2009; Murray et al., 2010). Before an intervention can be scaled up, it needs to make sense to the organization and the people implementing and the NPT framework helps provide this understanding (Hodge and Turner, 2016). Therefore, it is important to understand the interactions between contexts (organizational and technical structures), actors (providers, communities, and individuals), and objects (interventions, services, and procedures) and demonstrates how these factors can enable an intervention to become normalized or sustained in routine practice (May and Finch, 2009; Murray et al., 2010). The framework has four components: *coherence* (providers making sense of a practice, such as understanding the purpose of the parenting intervention and its potential value), *cognitive participation* (understanding the purpose, benefit, and the integration of the intervention into the daily work), *collective action* (resources and activities that enable implementation of a practice), such as having human resources or funding, and *reflexive monitoring* (the ongoing evaluation, feedback, and sense-making process that occur during and after implementation of the intervention). These components are understood to be in a dynamic process and promote providers’ understanding of, engagement with, and evaluation of the interventions to be integrated into routine practice. The NPT has previously been used to guide both the implementation of parenting support programs and analyses of the implementation process (Murray et al., 2010). In this study, we employ it as a conceptual framework to identify and understand the factors impacting the sustainability of the Ladnaan intervention in Swedish municipalities.

### The current study

Ladnaan is a parenting intervention that has been evaluated in the Swedish context with positive results. It consists of the Connect program (Moretti et al., 2009) and a societal information component (Osman et al., 2017a). The Connect parenting program is a manualized trauma- and attachment-focused parenting program intended to reduce children’s behavioral and social–emotional problems, family conflict and violence, and parental mental health problems while improving parent–child relationships and parents’ competence in parenting (Moretti et al., 2009). To ensure cultural sensitivity in the program, a qualitative study was conducted with Somali parents living in Sweden prior to the implementation of the Connect program. Based on the study findings, three topics of societal information were developed on the following topics (Osman et al., 2016): how Swedish Child Welfare Services function, particularly in relation to regulations regarding children’s placement in out-of-home care; the parenting system in the Swedish setting; and the Convention on the Rights of the Child (Osman et al., 2016). The Ladnaan intervention consists of 12 sessions (10 sessions of Connect program and two sessions of societal information) and is delivered in the parents’ mother tongue by group leaders with cultural and language competence. Each session included role-plays, reflection, and exercises, all of which were adapted to the parents’ cultural context. For instance, group leaders utilize examples that forcibly displaced people might face, as well as metaphors and proverbs to enhance parents’ understanding of specific concepts (Osman et al., 2022).

A randomized control trial showed that the program improved the mental health of parents and children and of parents’ efficacy and satisfaction in parenting (Osman et al., 2017b). These positive effects were maintained at a three-year follow-up (Osman et al., 2021b). In addition, parents who received the program reported that it helped them use more positive parenting practices and improve their relationships with their children (Osman et al., 2019). Several municipalities in Sweden have modified and implemented the Ladnaan intervention, and internationally, the Connect program has been implemented in culturally diverse contexts (e.g., Kenya, Italy, Mexico, and South Africa). However, a challenge has been scaling up and sustaining the program, particularly in Sweden and for forcibly displaced people (Osman et al., 2022). The current study aims to identify and understand the factors impacting the scalability and sustainability of the Ladnaan intervention in Swedish municipalities using NPT. Therefore, we ask the following research question: what are the common barriers and facilitators to scaling up and sustaining a parenting program for forcibly displaced people?

## Method

We used both quantitative and qualitative methods to obtain a comprehensive understanding of what facilitates the sustainable implementation of the Ladnaan intervention (Bryman, 2006; Creswell, 2013).

### Setting and implementation of the intervention

The study was conducted in three municipalities in Sweden, of which one was middle-sized (with a population of about 53,000 inhabitants), and the other two were small (with populations of about 12,000 and 5,000 inhabitants). These three municipalities were included in the study as they were implementing the Ladnaan intervention (i.e., both the Connect program and the societal intervention). The three municipalities varied in terms of when they started implementing parenting support programs for immigrant parents. However, at the onset of the current study, they all had a shared aim—namely, scaling up and sustaining the implementation of the Ladnaan intervention. The municipalities all had trained group leaders who could speak the parents’ mother tongues and were working within the municipalities. The intervention was delivered in Arabic, Dari, Kurmanji, Somali, Sorani, and Tigrinya.

Ethical approval was sought from the Swedish Ethical Review Authority (Dnr 2020-02748), which concluded that no ethical approval was needed for this study but shared an advisory opinion.

### Participants

Participants were recruited purposely using a snowball method and consisted of managers, coordinators, and group leaders involved in the implementation of the Ladnaan intervention. All participants provided oral informed consent. In total, two managers, six coordinators, and 11 group leaders participated in the study. They worked in social services (*n* = 5), integration offices (*n* = 4), schools or preschools (*n* = 7), or municipalities’ school or public health offices (*n* = 3). Of the 17 participants who specified their role in relation to the intervention, 10 (58.8%) directly delivered Ladnaan, five (29.4%) oversaw others delivering it, and two (11.8%) both delivered it directly themselves and oversaw others doing so.

### Data collection

**Figure 1:**
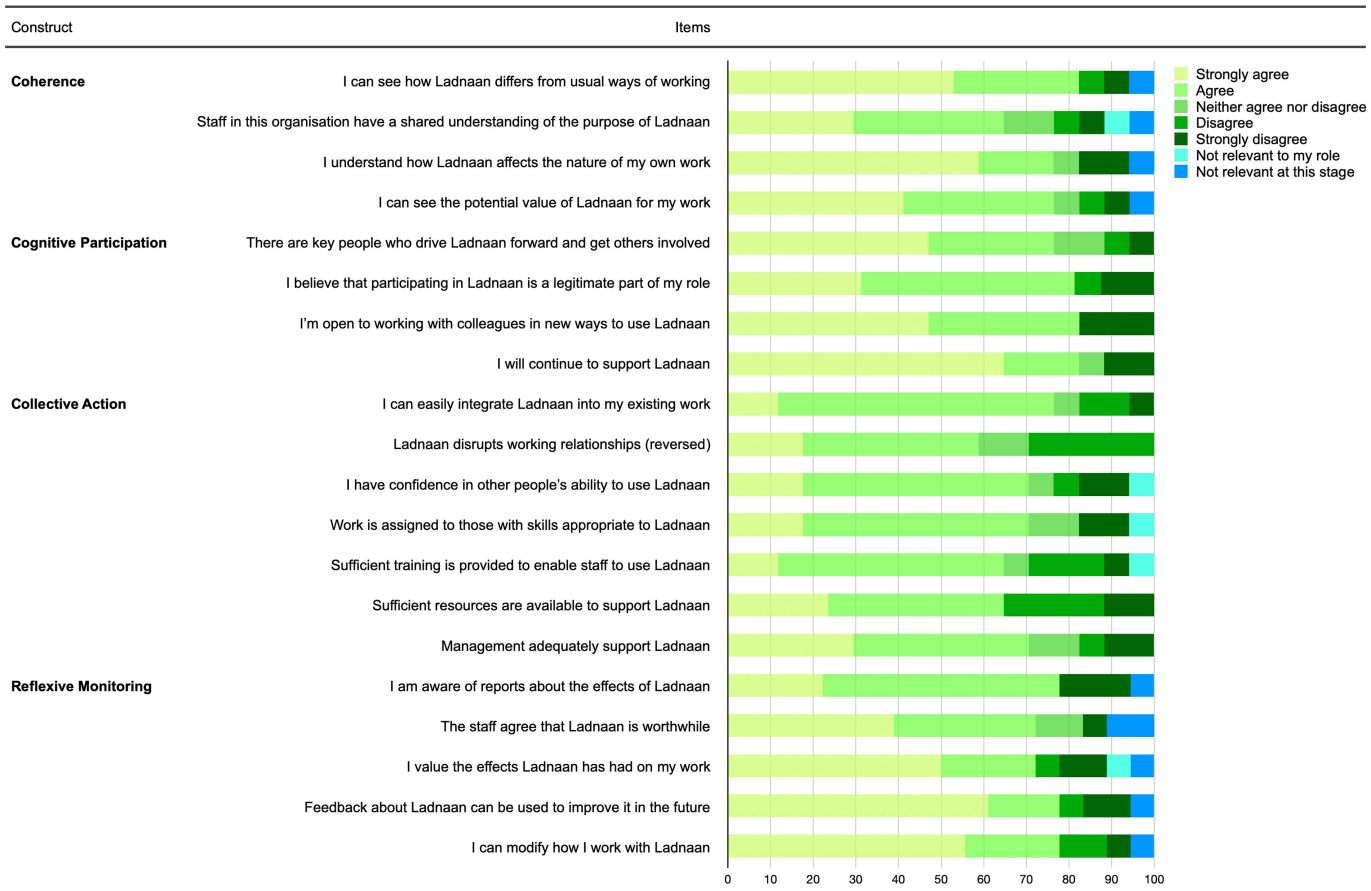
Distribution of response alternatives for each individual item in Normalisation Process Theory Measure (NOMAD).

The data, both quantitative and qualitative, were collected by the first author in 2020. For the quantitative data collection, the participants filled in the NPT Measure (NOMAD), which measures the intervention’s implementation from the perspective of NPT. NOMAD questionnaire has three sections: The first section (A) covers questions about the respondent (e.g., their role in their organization); the second (B) includes questions about their perception of the intervention in general, answered on a 10-point Likert scale; and the third (C) is divided into four sections covering the NPT themes: coherence, cognitive participation, collective action, and reflexive monitoring (see Figure 1). These items are answered on a 5-point Likert scale. Each participant completed the NOMAD questionnaire before the interviews or focus group discussions.

For the qualitative data, we conducted nine in-depth interviews and one focus group discussion with 10 participants. The interviews were with the managers and coordinators of the intervention, while we conducted the focus group discussions with the group leaders who delivered the intervention. We used a semi-structured interview guide for both the interviews and the focus group discussion. The interview guide covered particular themes to better understand what enabled or hindered the implementation of the Ladnaan intervention, as well as solutions or innovations that had been made during implementation. These questions were guided by the NPT components (see Table 1). The interview guide covered themes such as the need to implement the Ladnaan intervention within the municipality, barriers and facilitators to implementation (including organizational support, supervision and resources), and the contextual factors that may enhance scalability and sustainability. The interviews were conducted online or face-to-face, lasted 35–70 min, and were recorded with the participants’ consent. They were then assigned a code and transcribed verbatim.

**Table 1:** Use of NPT in the sustainable implementation of the Ladnaan intervention.

NPT components	Questions to consider within the NPT framework	Descriptive categories in the findings
**Coherence** *Making sense and understanding the Ladnaan intervention as a part of the interventions offered by the municipality*	- Do stakeholders (managers, facilitators and group leaders) have a shared sense of its purpose? - Do they share the benefits that the Ladnaan intervention brings to the immigrant families? - Is the Ladnaan intervention known in the municipality? - Is the Ladnaan intervention distinct from other interventions offered by the municipality?	- *Views on purpose*: need for a parenting program among immigrant families to enhance and strengthen their parenting practice - *Competes with other interventions:* the Ladnaan intervention was only known among professionals working with it
**Cognitive participation** *The collective work of committing to and engaging with the Ladnaan intervention as a part of new work practices*	- Do the stakeholders believe that they are the right people to drive the implementation forward? - Are the stakeholders aware of the people who could deliver and facilitate the implementation of the Ladnaan intervention? - Which organisation(s) has(ve) the potential to be engaged in the implementation of the Ladnaan intervention? - Are the stakeholders and municipality prepared to invest time, energy and work into it?	- *Engagement:* facilitators, managers and group leaders were the driving force behind the implementation of the program; challenges to recruiting group leaders were emphasised - *Ownership:* lack of ownership of the Ladnaan intervention and other parenting programs in the municipalities - *Leadership:* lack of ownership also led to a lack of leadership and maintenance of the program
**Collective action** *The work and resources stakeholders need to make the Ladnaan intervention function*	- What actions and plans have been made to scale up or have it embedded in routine practice? - Is there a need to train more group leaders to facilitate the implementation? - Are there resources and policies that support the implementation?	- *Resources:* lack of group leaders and motivation from the facilitators and managers jeopardised the scaling-up of the program - *Facilitator and not bound to a person:* binding the implementation to a person was a risk to scaling-up and sustaining the program - *Funding:* scaling-up the program depended on having a secure budget allocated to the implementation
**Reflexive monitoring** *Reflection on the appraisal and sustainability of the Ladnaan intervention*	- How do stakeholders and politicians perceive the intervention? - Is it clear what effects the Ladnaan intervention has had? - Can the Ladnaan intervention be sustained in the municipality? - Are there plans for and reflections on how sustainability can be ensured?	- *Use of evidence-based interventions:* different opinions on the use of evidence-based interventions - *Political decision:* importance of having a decision from politicians to secure the organisation and funding - *Saturation:* small municipalities might saturate their need quickly - *Municipality-wide comprehensive plan:* to guide the different interventions offered through the municipality

### Data analysis

Descriptive statistics were used to analyze the participants’ responses to the NOMAD questionnaire. Means and standard deviations were calculated to summarize each construct score, and frequencies were used to present the distribution of response alternatives for each individual item. The possible score range was 1 to 5, with higher scores indicating more successful implementation.

The transcribed interviews and focus group discussion were jointly analyzed using the qualitative-analysis-management software QSR N-Vivo version 11 (QSR International Pty, Ltd., Burlington, United States). The analysis was conducted in two steps: first, by taking an inductive approach and, second, a deductive approach (Hsieh and Shannon, 2005). The first step involved an inductive analysis in which the researchers sought patterns in the transcribed data. Each interview and the focus group discussion were read several times to get an overview of the participants’ experiences on what facilitated or hindered the scalability and sustainability of the parenting program. The text that captured those experiences was highlighted, and initial codes were developed. The first author was responsible for conducting the analysis. Once all the data were coded, the first author discussed the initial coding with all authors. All data were coded and then grouped according to their similarities and differences. A total of 12 descriptive categories were identified to describe the participants’ experiences in terms of what enabled or hindered the implementation of the Ladnaan program (third column of Table 1). The authors discussed these descriptive categories were agreed upon to carry forward the categories to the next step of the analysis.

The second step involved a deductive theory-driven analysis using the four themes of NPT. The analysis started by developing a matrix that included the four components of the NPT framework, along with guiding aligned with each component (see Table 1). All interview transcript and focus group discussions were read several times. The NPT questions helped in identifying data relevant to each of the four NPT framework components. Once the data were organized into the four components, we then compared with the categories derived from the inductive analysis. This comparison aimed to deepen understanding of how participants described the factors facilitated or hindered scaling up and sustaining the Ladnaan intervention. Finally, the results from both the inductive and deductive were condensed and integrated. In Table 1, we present the NPT themes, the NPT constructs/questions for this specific study, and the descriptive categories identified in the analysis.

## Results

The qualitative findings are presented within the NPT framework using the NPT constructs and study-specific questions (see Table 1). The means and standard deviations for each NPT construct domain are presented in Table 2.

**Table 2:** Descriptive statistics for each construct domain in NOMAD.

	n^a^	Mean	SD	Minimum	Maximum
Coherence	16	4.08	1.15	1.25	5.00
Cognitive participation	17	4.02	1.27	1.00	5.00
Collective action	16	3.56	0.92	1.57	4.43
Reflexive monitoring	17	4.06	1.25	1.40	5.00

NOMAD, Normalisation Process Theory Measure.

a Mean scores were calculated for participants with only one or no missing value at item level.

### Coherence

Participants scored high on the *coherence* responses to the NOMAD questionnaire, meaning that they understood the purpose and benefits of Ladnaan (see Figure 1). The findings from the focus group discussions showed that all participants shared a common perception and experience of the Ladnaan intervention’s main aims—namely, to enhance positive parenting, strengthen parent–child relationships, and improve immigrant families’ well-being. They agreed that the need to offer the Ladnaan intervention to immigrant families is as great as when the implementation started, if not greater.

Many participants mentioned that Ladnaan had certain additional benefits as it helped promote integration. They reported that after participating in the intervention, parents started taking more active roles in society, such as by organizing neighborhood or city walks to maintain safety in their communities.

*I think those who received the parenting program dare to do more or feel that they want to contribute to their society.* (Coordinator 2).

However, the participants also perceived that leaders and politicians in the municipalities lacked knowledge and understanding about the importance of having such an intervention embedded in practice. For example, one coordinator stated:

*The politicians might not understand the importance of having this intervention as part of a social worker’s job. And they complain about why there are problems. If I were a poor municipality, I would invest every day of the week in this intervention, both as an integration tool and to make parents confident and empowered.* (Coordinator 5).

The findings from the NOMAD responses showed that most participants were familiar with Ladnaan (range = 3 to 10, mean = 7.89, (standard deviation) [SD] = 1.91). However, according to some participants, not all professionals working in social services knew about Ladnaan or were familiar with delivering group-based interventions. They suggested that professionals should regularly receive information about interventions available to immigrant families (particularly culturally sensitive parenting programs) and their benefits.

The participants stressed that there was no coherence among professionals working in social services regarding evidence-based parenting support programs or about which programs and activities should be based on evidence. Although the Ladnaan intervention is distinct from other interventions, several participants noted that it competes with other interventions offered by the municipalities. For instance, in two municipalities, immigrant families were also provided with non-evidence-based parenting programs through educational unions and associations.

### Cognitive participation

The participants scored high on the *cognitive participation* responses. They felt that Ladnaan was currently a regular part of their work (range = 5 to 10, mean = 7.83, SD = 2.01) and would remain so in the future (range = 7 to 10, mean = 8.50, SD = 1.04). Most of the coordinators, managers, and group leaders believed that they were the right people to drive forward the implementation of the Ladnaan intervention but that coordinators in managerial positions were best positioned to successfully implement the work. This finding was evident in both the NOMAD responses and the focus group discussions.

*My opinion is to engage group leaders who have cultural competence in parents’ backgrounds and also of the host country. We become the bridge between the parents and the society.* (Male group leader—Focus group discussion).

*I agree with my colleague here; these parents were very afraid before they came to the sessions. For those I recruited they agreed because they trusted me* (Female group leader—Focus group discussion).

All participants agreed that group leaders with a background similar to that of the parents could most effectively facilitate the recruitment and engagement of immigrant families, but the small municipalities found it challenging to recruit and maintain such group leaders and suggested that interpreters could be used. One facilitator stated:

*When you do not have enough people with cultural and language competences, you have to find innovative ways to solve all the problems. For example, interpreters or trained group leaders from other municipalities could support the implementation.* (Coordinator 5).

To work collectively and engage all stakeholders in the process, coordinators and managers emphasized that there must be a clear sense of ownership within the municipality. Currently, the Ladnaan intervention is administratively housed under different departments in each municipality, which the respondents suggested is not sustainable.

*It is a fragmented responsibility … no given agency or stakeholder has the main responsibility … Is this something that schools can offer, the integration office can provide, or social services can offer?* (Coordinator 1).

*The structures are vague and weak, and the implementation of parenting programs is not statutory, and this is so important that it must be prioritized.* (Coordinator 6).

Coordinators and managers had different opinions about which organization was the best fit for the Ladnaan intervention and other parenting programs in terms of responsibility and sustainability. Some suggested that the responsibility should be assigned to an organization that parents and children encounter on a daily basis, such as schools or student health centers, while others suggested it should be assigned to an organization that deals with individuals whose needs are the greatest, such as social or integration services.

*Different needs for different purposes; if the need is to prevent, then it [Ladnaan] would be better placed in a school or integration office, but if the need is immediate, then social services are the ones with the competence.* (Manager 1).

Civil society organizations were also mentioned as important stakeholders—but as stakeholders without the resources to sustain the Ladnaan intervention without collaboration with social services. Collaboration within municipality departments and with other municipalities was emphasized as important for ensuring commitment to and engagement with the work of improving families’ well-being. Working with other departments and municipalities would decrease the burden on a single municipality—particularly small municipalities with smaller budgets. Group leaders felt that collaborating across departments and municipalities would lead to the scalability of the Ladnaan intervention.

*I live in [anonymized] municipality and work in this municipality as a group leader. So this means I am willing to move between municipalities, but are the municipalities willing to collaborate when it comes to delivering parenting programs to immigrant families?* (Female group leader—Focus group discussion).

Overall, there was a perceived lack of cognitive participation, mostly at the managerial and political levels, as the coordinators felt that the Ladnaan intervention and other parenting programs were not sufficient prioritized by politicians and frontline managers. The coordinators and managers emphasized that a decision at the political level is necessary to establish sustainable parenting programs. Another perceived hindrance was the uncertainty caused by the fact that governing politicians are replaced every 4 years. However, a municipality-wide comprehensive plan for parenting programs was suggested as a means of ensuring the sustainability of these programs, even when the political mandate changes.

### Collective action

Activities related to implementation continued in all the municipalities. In the middle-sized municipality, which started the implementation of Ladnaan in 2014, the program was delivered to two groups regularly each year, but only to Somali-born parents. The small municipality that started the implementation in 2018 offered Ladnaan in four languages in 2018 and 2019, serving eight groups per year. At the beginning of 2020, they started offering the intervention in two additional languages. In the third municipality, the implementation plans were delayed because of COVID-19.

The response distribution for all items in the *collective action construct* showed a lower score in all other constructs (see Figure 1). Participants did not see that Ladnaan was part of their work as many of them had other responsibilities. The NOMAD responses showed that there were not enough resources available for the effective scalability and sustainability of Ladnaan. Similar findings emerged from the focus group discussions.

*I have other responsibilities besides delivering the Ladnaan intervention, and sometimes, it is a challenge to know whether the municipality will continue with additional groups. It would have been good if there had been a group of people delivering the Ladnaan and the municipality hired them for a certain percentage, so we would not need to seek other jobs. The challenge is that if they do not think that way, they [municipalities] would need to train new group leaders everytime.* (Male group leader—Focus group discussions).

The participants highlighted that challenges to scaling up the intervention resulted from a lack of resources, finances, and motivation. Finding or retaining group leaders was difficult. To secure sustainability, two municipalities had trained their own existing workforce as group leaders. However, this strategy had not worked in the third municipality due to a shortage of personnel with necessary language and cultural competencies among the parents. Coordinators suggested that group leaders should be people from local authorities or schools who could work extra hours to deliver the intervention. Collaboration with other municipalities was mentioned as another solution. All participants emphasized the importance of having a facilitator to coordinate the implementation. At the same time, they noted that when the implementation work is bound to one person, it hinders the incorporation of the intervention into ordinary practice and its scalability and sustainability over time. The participants suggested that one organization could be responsible for implementing evidence-based parenting programs available to all parents in the municipality, but several departments could contribute to the funding.

A major challenge was the lack of finances to carry out the implementation and scaling up. The coordinators and managers stated that when no specific department in the municipality is responsible for the intervention, it becomes difficult to secure the necessary funding and resources for long-term intervention implementation. One facilitator stated:

*There should be a budget designated for parenting programs; we have funding for three years, which makes our implementation work easier. But then we do not know what happens afterwards* (Coordinator 3).

### Reflexive monitoring

Although the NOMAD responses showed high scores in all the items *reflexive monitoring* construct, they scored lower in their awareness of the effects of Ladnaan intervention. This suggest that their knowledge of the most current evidence concerning parenting programs was limited, indicating a lack of awareness regarding the research-based knowledge encompassing parenting programs in general and specifically, the Ladnaan intervention.

The findings from the interviews highlighted differences among coordinators and managers regarding the implementation of evidence-based programs. Most coordinators agreed on the importance of using evidence-based programs, such as Ladnaan, to improve immigrant families’ well-being. However, other respondents stated that evidence-based parenting programs are more expensive to use and require regular updates, which may hinder their sustainability. Nonetheless, several coordinators emphasized that interventions provided to parents should be fundamentally based on evidence. It was also agreed that a systematic evaluation should be in place to which other stakeholders might contribute.

*We who are working in the social services should think of using methods that have been tested and proven to have an effect.* (Coordinator 1).

Respondents from the two small municipalities mentioned that while the need for an evidence-based parenting support program in the municipality had been temporarily fulfilled, it was important to maintain the capacity to offer these programs and also to consider offering more individualized initiatives. A facilitator in one of the municipalities stressed this:

*We are such a small municipality, and we have delivered [the program] to many parents. However, many parents have moved due to relocation by the migration office, and people move to other cities too. We will stop here and reflect on how we can develop further and how we can still offer support to immigrant families.* (*Coordinator* 2).

## Discussion

In this study, we aimed to identify and understand the factors impacting the sustainability of a culturally tailored parenting program in Swedish municipalities. More specifically, we used NPT to explore the context, actors, and objects as factors promoting the sustainable implementation of the Ladnaan intervention. Our quantitative and qualitative analyses were consistent. They showed that participants had a common understanding of the benefits of delivering the Ladnaan intervention to forcibly displaced families. However, a lack of ownership, resources, and financing were common problems that hindered the sustainability of not only the Ladnaan intervention but also any other evidence-based social services.

Previous studies have shown that forcibly displaced families express the need for culturally sensitive parenting support programs to strengthen their parenting, integration, and mental well-being (Baghdasaryan et al., 2021; Osman et al., 2016). The managers and coordinators participating in this study expressed a similar view and showed coherence on the importance of culturally tailored parenting programs as targeted interventions to promote refugee families’ well-being. However, they also believed that their frontline managers and municipality politicians did not share this view. Due to limited engagement at the managerial and political levels, municipalities did not have a comprehensive plan to ensure the long-term availability of parenting programs. The scalability of parenting programs delivered to immigrant or disadvantaged groups is supported by the engagement of leaders at the municipality and continuous support for implementers, which facilitates the collective work (Hodge et al., 2017).

The recruitment and retention of group leaders was mentioned as one of the main challenges in sustaining Ladnaan as the number of forcibly displaced families and the need for the program fluctuate over time. Weeks (2021) indicated that group leaders’ buy-in positively contributes to the improved scalability and sustainability of parenting interventions. The level of engagement, belief, and commitment to a program’s content influences the success of the program’s implementation and its impact on the parents who receive it (Martin et al., 2023; Salomone et al., 2022). Offering group leaders continuous development could help in both retaining them and ensuring their adherence to the program (Martin et al., 2023; Weeks, 2021). It is important to acknowledge the challenges that small municipalities may face in sustaining any targeted intervention. The need for such interventions can vary greatly over time, and sufficient resources may not be available to monitor the need and sustain the capacity to offer a variety of services to a small population.

The few existing studies on the sustainability of evidence-based parenting programs in social services suggest that stakeholders’ engagement and collaboration with the community may bolster sustainability (Hodge and Turner, 2016; Hodge et al., 2017; Shelton et al., 2018; Weeks, 2021). However, for such collaboration and partnership to work, previous studies have found that it is crucial to have a coordinator or program champion who serves as the motivator or advocate for the program and facilitates coordination among the various actors involved in the implementation (Ericsson and Sarkadi, 2021; Hodge and Turner, 2016; Shelton et al., 2018). We observed the same need in our study.

The participants in our study stated that there was a lack of awareness among social workers regarding the Ladnaan intervention. Insufficient awareness of available services is a common problem as social workers might fail to offer forcibly displaced parents tailored interventions that exist within the municipality. Future research should explore whether co-creating interventions with service providers and users can lead to increased awareness and sustainable program implementation over time (Knutsson and Schön, 2020). Additionally, there is a need to investigate whether, after receiving cultural sensitivity in-staff training, staff who do not share parents’ immigrant background can deliver the program as effective as staff from the same background.

Incorporating scalability and sustainability strategies at the beginning of program implementation has been shown to be crucial for the sustainability of evidence-based programs in social services (Ghate, 2016; Shelton et al., 2018). This might be a challenge when an intervention is implemented by the researchers, and the municipality is not engaged before the funding is applied. Therefore, it is important to work in partnership with the municipality or stakeholders to implement as this may enhance the scalability and sustainability of parenting programs delivered to immigrant families (Hodge and Turner, 2016). Our study demonstrates that these strategies need to be regularly revisited and revised.

### Limitations

Although our study contributes to the understudied area of the scalability and sustainability of parenting programs for forcibly displaced parents within social services, it contains some methodological limitations. One limitation is that all three municipalities included in the study were relatively small and in rural and suburban areas; hence, the findings from this study cannot be generalized to larger or urban municipalities. Another limitation concerns the sample. The participants, who were recruited through convenience sampling, worked with the implementation and delivery of parenting programs in local governmental organizations. There is also a risk for social desirability bias. Participants may have emphasized external facilitators and barriers instead of addressing interpersonal barriers. We lacked the voices of leaders and politicians at the municipality level, who could have contributed their experiences and views on the factors involved in establishing sustainable evidence-based programs at the local level. However, the study’s aim was not to achieve generalizability but rather to examine the different factors that may contribute to the implementation and sustainability of Ladnaan in social services. Interviews were conducted both face-to-face and online, with the choice of location made by the participants. This may explain why one online interview lasted only 30 min. However, the data from this interview was rich, and we cannot be sure how it would have differed if it had been conducted face-to-face.

## Conclusion

The findings from this study illustrate the complexity of scaling up and sustaining parenting programs for forcibly displaced people, such as Ladnaan, in real-world settings—particularly in social services and small municipalities. Our findings show that three of the key components of the NPT framework that promote the integration of interventions into routine practice—coherence, cognitive participation, and reflective monitoring—were in place, as reported by participants. However, one key component, collective action, which refers to the resources and activities that enable sustainability, was lacking. The findings indicate that insufficient leadership and ownership by local authorities hindered the sustainability of all parenting programs. For instance, the organization that held responsibility for implementing the Ladnaan program in local settings differed among the municipalities included in this study, which was considered a challenge to the program’s sustainability. The findings suggest that engaging and collaborating with local authorities and civil society might contribute to maintaining the long-term implementation of such programs. This partnership might also mitigate the complexity around sustainability, such as the recruitment of program providers (coordinators and group leaders). Our study highlights the importance of promoting culturally tailored parenting programs in schools, social services, civil society and other services available for forcibly displaced people. It also emphasized the importance of coordinating efforts to support families and fostering collaboration through co-creation between social services and civil society.

## Data Availability

The datasets presented in this article is not readily available. In order to make it available, approval must be sought from the Swedish Ethical Review Authority. Requests to access the datasets should be directed to https://www.etikprovningsansokan.se/.
